# Analysis of Notch1 and Notch3 Signaling Pathway Components in Benign Prostatic Hyperplasia and Prostate Cancer Patients

**DOI:** 10.1002/jcla.70339

**Published:** 2026-08-18

**Authors:** Emine Yagci, Cansu Ozbayer, Ata Ozen, Gamze Zengin, Hulyam Kurt

**Affiliations:** ^1^ Department of Medical Biology Kutahya Health Sciences University, Faculty of Medicine Kutahya Turkey; ^2^ Department of Urology Eskisehir Osmangazi University, Faculty of Medicine Eskisehir Turkey; ^3^ Department of Medical Biology Eskisehir Osmangazi University, Faculty of Medicine Eskisehir Turkey

**Keywords:** genetic polymorphism, notch signaling pathway, NOTCH1, NOTCH3, prostate cancer, serum protein levels

## Abstract

**Background:**

Prostate cancer (PCa) and benign prostatic hyperplasia (BPH) significantly affect men's quality of life. Due to low PSA specificity, more sensitive genetic and protein markers are needed. This study comparatively evaluates variations in NOTCH1 and NOTCH3 gene regions—key components of the Notch pathway—and serum protein levels in PCa and BPH patients.

**Methods:**

The study included 90 individuals: 15 healthy controls, 25 BPH, 25 localized PCa (LPCa), and 25 metastatic PCa (MPCa) patients. NOTCH1 and NOTCH3 gene regions were analyzed via Sanger sequencing, and serum protein levels were measured using ELISA.

**Results:**

Genetic analyses revealed that the novel NOTCH1 g.54305 T>A variation (*p* = 0.0001), rs2133314984 (*p* = 0.015), and rs577451694 (*p* = 0.0012) variants were significantly associated with increased PCa risk. Additionally, the NOTCH3 rs746192089 variation was linked to disease risk (*p* = 0.0005), whereas the rs1044009 variant showed no statistically significant difference. Protein assessments showed that serum NOTCH1 concentrations were significantly lower in LPCa (*p* = 0.006) and MPCa (*p* = 0.003) groups than controls. Serum NOTCH3 levels were significantly decreased (*p* ≤ 0.002) in BPH, LPCa, and MPCa groups, with the lowest levels in the metastatic stage. Wild‐type NOTCH1 g.54305 TT and NOTCH3 rs746192089 CC genotypes were associated with significantly higher serum protein expression than mutant‐allele genotypes (*p* = 0.036 and *p* < 0.001, respectively).

**Conclusions:**

In conclusion, specific NOTCH1 and NOTCH3 variations are linked to disease risk, and decreased serum protein levels correlate with advanced clinical stages, providing insights into the Notch pathway's role in PCa pathogenesis.

## Graphical abstract

This study evaluates the association between Notch1 and Notch3 genetic variations and serum protein levels in patients with benign prostatic hyperplasia and prostate cancer. While genetic variations showed no significant link to disease risk, serum protein levels were significantly lower in prostate cancer and metastatic groups compared to healthy controls. These findings suggest that the suppression of the Notch signaling pathway is linked to disease progression, supporting the potential use of these proteins as biomarkers for diagnosing and monitoring prostate pathologies.

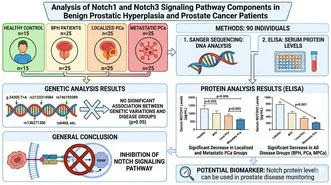

## 1 Introduction

Prostate cancer (PCa) is one of the most common and significant malignancies threatening men's health within the global oncology burden. According to the 2025 data, PCa accounts for 30% of cancer cases in men and ranks second in cancer‐related deaths after lung cancer [1]. Similarly, benign prostatic hyperplasia (BPH) is creating an increasing health burden in clinical practice due to the aging population and changing lifestyle factors. BPH is characterized by lower urinary tract symptoms, particularly urethral obstruction, and leads to a significant decrease in patients' quality of life [2]. Both pathologies arise from the disruption of the proliferative balance in the prostate epithelium; however, distinguishing the molecular basis of these processes, particularly their metastatic potential, is of critical importance for the development of targeted diagnostic and therapeutic strategies.

Prostate carcinogenesis has a complex relationship with genetic mutations, androgen receptor signaling pathways, and alterations in testosterone metabolism [3]. In this context, the Notch signaling pathway, which determines cell fate and regulates tissue homeostasis, emerges as a key factor in understanding prostate abnormalities. Notch signaling has a dual nature, capable of exerting oncogenic or tumor‐suppressive roles depending on the tissue type and biological context. For example, it exhibits oncogenic activity in T‐cell acute lymphoblastic leukemia, whereas it may function as a tumor suppressor in myeloid malignancies [4].

Notch receptors constitute a highly conserved family of single‐pass transmembrane proteins that are widely expressed in higher organisms, and canonical Notch signaling plays a critical role in regulating cell fate, proliferation, differentiation, and apoptosis through cell–cell interactions [5]. In mammals, the Notch signaling pathway consists of four homologous receptors (NOTCH1–NOTCH4) and five ligands, including three Delta‐like proteins (DLL‐1, DLL‐2, and DLL‐4) and two Jagged proteins (Jagged‐1 and Jagged‐2) [6].

In prostate tissue, aberrant Notch signaling is known to trigger carcinogenesis by affecting cell adhesion and invasive capacity [7]. In particular, the critical role of the Notch1 receptor in prostate organogenesis and bud formation suggests its central involvement in cancer progression. Indeed, the literature demonstrates that inhibition of Notch1 can suppress invasion in prostate cancer cells and enhance chemosensitivity. On the other hand, Notch3, while being essential for luminal cell differentiation during normal prostate development, has been reported to show a strong association between its high expression and cancer recurrence, drug resistance, and, notably, bone metastasis and angiogenesis [8, 9].

Although biomarkers such as PSA are widely used in current clinical practice, the low specificity in distinguishing BPH from PCa and the risk of overdiagnosis have increased the need for more specific genetic‐ and protein‐based markers. While studies in the literature have largely focused on protein expression, the impact of genetic variations (SNPs) within the genes encoding these receptors on serum protein levels and disease stages has not yet been fully elucidated.

The aim of this study is to analyze the Notch1 and Notch3 gene regions using Sanger sequencing; to comparatively evaluate the relationship between genetic variations and serum protein levels in PCa and BPH patients; and to investigate the potential biomarkers of these molecules.

## 2 Material and Methods

### 2.1 Investigated Group

The investigated group consisted of 15 control subjects and 75 patients who were diagnosed with benign prostatic hyperplasia (BPH, *n* = 25), localized prostate cancer (LPCa, *n* = 25), and metastatic prostate cancer (MPCa, *n* = 25) of Turkish origin and were recruited from the Department of Urology, Eskisehir Osmangazi University, Faculty of Medicine in Eskisehir, Turkey.

All patients in the BPH, LPCa, and MPCa groups included in the study were selected from treatment‐naive cases. According to the clinical protocol, all patients were newly diagnosed and had not received any systemic/local therapeutic regimens such as androgen suppression therapy (ADT), chemotherapy, or radiotherapy, nor had they undergone any surgical intervention before peripheral blood samples were collected. Detailed data on the demographic and clinical characteristics of the participants are presented in Table 1.

**TABLE 1:** Demographic and clinical characteristics of the study groups.

Parameters	Control (*n* = 15)	BPH (*n* = 25)	LPCa (*n* = 25)	MPCa (*n* = 25)	*p*
Age (years)	32.6 ± 4.1	70.7 ± 8.2	68.3 ± 7.6	70.9 ± 11.2	**< 0.001** ^a^
PSA	NA	3.98 (0.39–15.9)	13.9 (5.1–55.0)	157.0 (14.9–8616.0)	**< 0.001** ^b^
ISUP (Gleason)	NA	NA	2 (1–5)	4 (1–5)	**< 0.001** ^c^

*Note:* Age data are presented as Mean ± Standard Deviation, and PSA and ISUP data as Median (Minimum–Maximum). Bold *p*‐values indicate statistical significance.

^a^ One‐way ANOVA test (control group significantly different from the other 3 groups).

^b^ Kruskal–Wallis test (all 3 groups statistically significantly different from each other).

^c^ Mann–Whitney *U* test (only LPCa and MPCa groups were compared).

The mean age of the control group (32.67 ± 4.08 years) was lower compared to all other patient groups with clinical diagnoses (*p* < 0.001). In contrast, the groups in the active disease spectrum (BPH, LPCa, and MPCa) had a homogeneous age distribution, and there was no statistically significant difference in mean age between these three groups (*p* > 0.05).

The range of the control group is a conscious methodological choice based on established epidemiological data. In the literature, Benign Prostatic Hyperplasia is considered an inevitable physiological process in the aging male population, and prevalence rates are reported to reach approximately 80% by the age of 80 [10, 11]. Given the difficulty of clinically excluding subclinical or asymptomatic prostate enlargement in older men, a younger control cohort was planned to obtain a more biologically pure and reliable healthy baseline free from contamination. This strategic approach was designed to minimize potential age‐related confounding effects and to establish a stronger, clearer biochemical distinction between healthy physiological states and prostate pathologies.

When serum PSA levels, one of the basic clinical and laboratory parameters of the cases, were evaluated, a statistically significant difference was found between the diagnostic groups (*p* < 0.001). The median PSA value of the MPCa group, representing advanced stage and widespread disease burden, was 157 ng/mL. Serum PSA levels were found to be significantly higher in both the LPCa group with localized tumor burden (13.9 ng/mL) and the BPH group with benign pathology (3.98 ng/mL). Similarly, serum PSA levels in patients in the LPCa group were also found to be significantly higher than in cases in the BPH group.

When the ISUP (Gleason) grade groups, reflecting the histopathological and clinical aggressiveness of cases diagnosed with malignancy, were examined, it was determined that the median tumor grade (Grade 4) of metastatic patients (MPCa) was statistically significantly higher and exhibited a more aggressive clinical phenotype compared to patients with localized tumors (LPCa, Grade 2) (*p* < 0.001).

### 2.2 DNA Isolation and Quantitation

Peripheral blood and serum samples collected from patients and healthy volunteers were used for DNA isolation. DNA was isolated from the blood samples of patients and control individuals via the GeneMATRIX Quick Blood DNA Purification Kit (EURx, Poland) following the manufacturer's protocol. DNA purity analysis of all the isolated samples was performed spectrophotometrically with a Nano500 device (Allsheng, Hangzhou, China). The A260/A280 ratios of all the DNA samples were measured, and reisolation was performed for samples whose A260/A280 ratios were less than 1.7. After 1.7 ≤ A260/A280 ≤ 2.0 was achieved for all our DNA samples, the DNA stock concentration was diluted with elution solution to 50 ng/μL and stored at −20°C until the genotyping stage.

The NCBI and Ensembl databases were used to select the sequencing region to be investigated within the scope of our study. In this context, 530 bp and 952 bp regions were selected for the NOTCH1 and NOTCH3 genes, respectively. Targeted sequencing regions for NOTCH1 and NOTCH3 were identified based on specific genomic coordinates using RefSeqGene arrays. For NOTCH1, a 530 bp region located in exon 34 between bases 54,219 and 54,748 of the reference sequence NG_007458.1 was amplified. For NOTCH3, a 952 bp region located in exon 33 between bases 44,303 and 45,254 of the reference sequence NG_009819.1 was targeted.

Primers were designed for the amplification of these regions, and the following primer sequences were synthesized:

NOTCH1‐Forward: 5′‐CAGATGCAGCAGCAGAACCTG‐3′.

NOTCH1‐Reverse: 5′‐AAAGGAAGCCGGGGTCTCGT‐3′.

NOTCH3‐Forward: 5′‐AAGGGAGCTGGGAACAGAC‐3′.

NOTCH3‐Reverse: 5′‐CAGCTATGAGGCTGCCAAG‐3′.

Gradient PCR was performed for amplification. The optimized PCR conditions and PCR mixture for amplification were as follows: 4 μL of FIREPol Master Mix 5X (Solis BioDyne, Europe), 0.5 μL of each primer (10 nm), 1 μL of DNA template, and 14 μL of nuclease‐free water. PCR was conducted as follows: denaturation at 94°C for 3 min; 30 cycles of 94°C for 30 s, 58.4°C for Notch1 or 66.5°C for Notch3 for 1 min and 72°C for 1 min; and a final elongation at 72°C for 5 min.

### 2.3 Purification and Sequencing

After the isolated DNA samples were amplified via polymerase chain reaction with primers and PCR mix, HighPrep PCR Purification was performed on approximately 530 bp long amplicons, which were subsequently genotyped via one‐way sequencing (SANGER sequencing). Sequence analysis of the samples was carried out one way (forward) via specific primers used in the PCR process.

DNA sequencing was performed via the ABI 3730XL Sanger sequencing device (Applied Biosystems, Foster City, CA) and the Big Dye Terminator v3.1 Cycle Sequencing Kit (Applied Biosystems, Foster City, CA), and as a result, raw DNA sequences were obtained.

The resulting chromatogram files were exported with SNAPGene Viewer and aligned with MEGA11 software. Sequence data were blasted via the NCBI (National Center for Biotechnology Information) BLAST database.

### 2.4 ELISA Analysis

Serum samples were aliquoted immediately after collection, in single‐use portions for each specific protein, and stored at −80°C for a maximum of 12 months until analysis. Samples were thawed only once during analysis, completely avoiding repeated freeze–thaw cycles to prevent pre‐analytical protein degradation. Serum Notch1 and Notch3 levels were measured using commercial ELISA kits (Uscn Life Science Inc., Wuhan, China) and read at 450 nm wavelength using a Multiskan GO Microplate Spectrophotometer (Thermo Scientific). Protein concentrations were calculated using four‐parameter logistic (4PL) curve fitting derived from the standard curve.

According to manufacturer data, the detection range for both kits was 0.156–10 ng/mL. Minimum detectable doses were determined as < 0.060 ng/mL for Notch1 and < 0.057 ng/mL for Notch3. Intra‐assay and inter‐assay coefficients of variation (CV) were recorded as < 10% and < 12%, respectively. To manage outliers, samples with an inter‐replica CV greater than 15% were re‐analyzed. To control for pre‐analytical factors that could affect serum protein measurements, blood samples were collected under strictly standardized fasting conditions, and samples that were visibly lipemic or hemolyzed were excluded from the analysis.

### 2.5 Statistical Analysis

Statistical analysis of the data obtained from the study was performed using IBM SPSS Statistics 27 and Sigma Stat 3.5 software packages. In all analyses, a *p* < 0.05 value was accepted as the statistical significance threshold. The normality of age, PSA, and ISUP grade group data was evaluated using the Shapiro–Wilk test. Age data were expressed as Mean ± Standard Deviation, while PSA and ISUP grade group data (which did not show a normal distribution) were expressed as Median (Minimum–Maximum). One‐Way Analysis of Variance (ANOVA) was used to compare the mean ages of the four groups, and the Tukey HSD test was used for post hoc analyses. The Kruskal–Wallis test was used to compare PSA levels between the BPH, LPCa, and MPCa groups, and the Dunn test was used for post hoc multiple comparisons. The Mann–Whitney *U* test was used to compare the tumor grades (ISUP grade group) of the LPCa and MPCa groups. In the scope of genetic analyses, the allele distributions and frequencies of the variations detected in the NOTCH1 and NOTCH3 gene regions were determined. Pearson chi‐square test was used to compare genotype distributions between case and control groups. Hardy–Weinberg equilibrium was evaluated for each variant in control and patient groups using the chi‐square goodness‐of‐fit test. In genetic association analyses, allelic, dominant, recessive, heterozygous, and homozygous inheritance patterns were examined; odds ratio (OR), 95% confidence interval (CI), and *p*‐values were calculated. In addition, the Cochran–Armitage trend test was applied to evaluate the genotype‐dose effect. In the comparative analysis of serum Notch1 and Notch3 protein levels between groups, the Mann–Whitney rank‐sum test, a non‐parametric test, was used after evaluating the suitability of the data to a normal distribution. Data on protein levels were expressed as mean ± standard deviation (Mean ± SD) values, and the results were supported by figures. Kruskal–Wallis and Mann–Whitney *U* tests were used to compare protein levels according to genotypes in the variants examined.

The study's post hoc power analysis (GPower 3.1.9.7) was performed based on serum Notch1 and Notch3 protein levels, the primary endpoints showing significant differences between groups. In the analysis, conducted with a total of 90 cases and an alpha = 0.05 significance level, the effect size (Cohen's *f*) for Notch1 protein levels was found to be 1.672 and the statistical power was 100% (1.00). For Notch3 protein levels, the effect size (Cohen's *f*) was calculated as 2.786 and the statistical power was 100% (1.00). These results confirm that the study's sample size is highly sufficient to reveal biological differences between groups and that the study has excellent statistical power, well above the internationally accepted 80% power threshold.

## 3 Results

Within the scope of our study, prostate cancer risk and Notch1 and Notch3 gene regions and their serum levels were investigated and the obtained data are presented in this section.

### 3.1 Notch1 Gene Variations

#### 3.1.1 Position 54,305 Variation

Bioinformatic analyses of the 34th exon sequence of the NOTCH1 gene (NM_017617.5) revealed a novel nucleotide variation (g.54305 T>A) not previously reported in the literature and not yet identified in the dbSNP (v157) database (Material S1). NCBI BLAST alignment analysis presented the query sequence and reference genomic region along with neighboring SNPs obtained from the dbSNP database. However, it was confirmed that this specific variant at position 54,305 is not included in the dbSNP v157 database and is identified for the first time in this study (Material S1). Statistical analysis revealed a significant relationship between the genotype frequency distributions of the Notch1 Position 54,305 variation and the groups (Control, BPH, LPCa, and MPCa) (*X* ^2^ = 26.89, *p* < 0.001). In the control group, the most common genotype was the homozygous TT genotype (80%, *n* = 12), while this rate gradually decreased as the disease stage progressed, falling to 12% in the LPCa group (*n* = 3) and 16% in the MPCa group (*n* = 4). The homozygous variant, the AA genotype, was observed at a rate of 6.7% in the control group; however, it increased to 20% in the BPH group, 44% in the LPCa group, and 36% in the MPCa group. The heterozygous TA genotype showed a similar and high frequency distribution in the BPH (52%), LPCa (44%), and MPCa (48%) groups (Table 2).

**TABLE 2:** Genotype distribution and characteristics of Notch1 and Notch3 gene variants.

Notch1 gene variants									
	Position 54,305			rs2133314984			rs577451694		
	TT	TA	AA	TT	TA	AA	GG	GC	CC
Control	12	2	1	15	—	—	14	1	0
BPH	7	13	5	24	1	—	17	1	7
LPCa	3	11	11	20	5	—	18	2	5
MPCa	4	12	9	20	5	—	12	0	13
Statistics	***X*** ^**2**^ **= 26.89, *p* < 0.001**			*X* ^2^ = 6.483, *p* = 0.0903			***X*** ^**2**^ **= 14.946, *p* = 0.0207**		

Notch3 gene variants						
	rs746192089			rs1044009		
	CC	GC	GG	CC	TC	TT
Control	14	1	—	0	5	10
BPH	11	8	6	3	10	12
LPCa	7	13	5	3	7	15
MPCa	2	17	6	4	8	13
Statistics	***X*** ^**2**^ **= 31.891, *p* < 0.0001**			*X* ^2^ = 3.536, *p* = 0.7392		

*Note:* Bold values indicate statistically significant.

The genotype distributions for the g.54305 T>A variation were found to be consistent with Hardy–Weinberg equilibrium (HWE) in both the non‐cancerous control group (*p* = 0.64) and the prostate cancer group (*p* = 0.97). When allele frequencies were compared, the frequency of the A allele was found to be significantly higher in the prostate cancer group (63.0%) than in the non‐cancerous group (33.8%) (*p* = 0.0001; OR = 3.34, 95% CI: 1.74–6.42). Similarly, a statistically significant difference was found between the two groups in terms of overall genotype distributions (TT, TA, AA) (*X* ^2^ = 10.16, *p* = 0.006; Table 3).

**TABLE 3:** Genotype and allele frequencies of Notch1 Position 54,305, rs2133314984, rs577451694 variations in the prostate cancer (LPCa and MPCa) group and non‐cancerous (BPH and control) group.

SNP position 54,305	Allele		Statistic *p*	OR (95% CI)	Genotype			Statistic *p*	HWE
	T *n* (%)	A *n* (%)			TT	TA	AA		
Non‐cancerous	53 (66.2)	27 (33.8)	**0.0001**	3.34 (1.74–6.42)	19	15	6	***X*** ^*2*^ **= 10.16** ***p* = 0.006**	0.64
Prostate cancer	37 (37.0)	63 (63.0)			7	23	20		0.97
OR (95% CI) (Risk allele A)									
Heterozygous TT vs. TA		Homozygous TT vs. AA		Dominant TT vs. TA + AA		Recessive TT + TA vs. AA		Armitage's trend test	
2.77 (0.91–8.39) *p* = 0.06		9.03 (2.58–31.6) ***p* = 0.0005**		5.55 (1.85–16.7) ***p* = 0.002**		3.78 (1.19–11.9) ***p* = 0.02**		***X*** ^*2*^ **= 13.9** ***p*** **= 0.002**	

SNP rs2133314984	Allele		Statistic *p*	OR (95% CI)	Genotype			Statistic *p*	HWE
	T *n* (%)	A *n* (%)			TT	TA	AA		
Non‐cancerous	79 (98.8)	1 (1.2)	**0.015**	8.78 (1.19–79.8)	39	1	0	***X*** ^*2*^ **= 6.35** ***p* = 0.012**	0.98
Prostate cancer	90 (90.0)	10 (10.0)			40	10	0		0.87
OR (95% CI) (Risk allele A)									
Heterozygous TT vs. TA		Homozygous TT vs. AA		Dominant TT vs. TA + AA		Recessive TT + TA vs. AA		Armitage's trend test	
9.75 (1.19–79.8) ***p* = 0.02**		N/A		9.75 (1.19–79.8) ***p* = 0.02**		N/A		***X*** ^*2*^ **= 5.8** ***p*** **= 0.016**	

SNP rs577451694	Allele		Statistic *p*	OR (95% CI)	Genotype			Statistic *p*	HWE
	G *n* (%)	C *n* (%)			GG	GC	CC		
Non‐cancerous	64 (80.0)	16 (20.0)	**0.0012**	2.45 (1.24–4.83)	31	2	7	*X* ^*2*^ = 3.79 *p* = 0.15	< 0.001
Prostate cancer	62 (62.0)	38 (38.0)			30	2	18		< 0.001
OR (95% CI) (Risk allele C)									
Heterozygous GG vs. GC		Homozygous GG vs. CC		Dominant GG vs. GC + CC		Recessive GC + GG vs. CC		Armitage's trend test	
1.03 (0.13–8.2) *p* = 0.97		2.66 (1.00–7.07) *p* = 0.05		2.30 (0.87–6.09) *p* = 0.09		2.65 (1.02–6.89) ***p* = 0.04**		***X*** ^*2*^ **= 11.2** ***p*** **= 0.0008**	

*Note:* Bold values indicate statistically significant.

In genetic modeling based on the A risk allele, when odds ratios (OR) were evaluated, it was found that the AA genotype was highly and significantly associated with prostate cancer in the homozygous model (OR = 9.03, 95% CI: 2.58–31.6, *p* = 0.0005). In the dominant model, carrying either the TA or AA genotype was found to significantly increase the risk of cancer (OR = 5.55, 95% CI: 1.85–16.7, *p* = 0.002). In the recessive model, the AA genotype was found to have a significant association with prostate cancer risk (OR = 3.78, 95% CI: 1.19–11.9, *p* = 0.02). Although the heterozygous model approached statistical significance, no strictly significant difference was observed (OR = 2.77, 95% CI: 0.91–8.39, *p* = 0.06). Finally, the Armitage trend test confirmed a statistically significant trend between variant genotypes and prostate cancer risk (*X* ^2^ = 13.9, *p* = 0.002; Table 3).

#### 3.1.2 rs2133314984 Variation

As a result of BLAST alignment performed within the scope of bioinformatics analysis, the rs2133314984 variant of the NOTCH1 gene was found to be located in exon 34. The obtained NCBI BLAST alignment output shows the location of the query sequence and the reference genomic region, as well as the placement of neighboring SNPs obtained from the dbSNP database (Material S2).

The genotype frequencies of the Notch1 rs2133314984 variation were examined according to the distribution in the study groups, and no statistically significant relationship was found between the groups (*X* ^2^ = 6.483, *p* = 0.0903). Homozygous TT genotype was detected in all individuals in the control group (100%, *n* = 15). The frequency of heterozygous TA genotype was determined as 4% (*n* = 1) in the BPH group, 20% (*n* = 5) in the LPCa group, and 20% (*n* = 5) in the MPCa group. The AA genotype was not found in any study group (Table 2).

Genotype distributions for the rs2133314984 variant were found to be consistent with Hardy–Weinberg equilibrium (HWE) in both the non‐cancerous control group (*p* = 0.98) and the prostate cancer group (*p* = 0.87). When allele frequencies were compared, the frequency of the A allele was found to be significantly higher in the prostate cancer group (10.0%) than in the non‐cancerous group (1.2%) (*p* = 0.015; OR = 8.78, 95% CI: 1.19–79.8). A statistically significant difference was also found between the two groups in terms of overall genotype distributions (TT, TA, AA) (*X* ^2^ = 6.35, *p* = 0.012). The AA genotype was not found in either group (Table 3).

When Odds ratios (ORs) were evaluated in genetic models based on the A risk allele, a statistically significant association was found between the TA genotype and prostate cancer risk in the heterozygous model (OR = 9.75, 95% CI: 1.19–79.8, *p* = 0.02). Similarly, in the dominant model, it was found that carrying the TA or AA genotype significantly increased the cancer risk (OR = 9.75, 95% CI: 1.19–79.8, *p* = 0.02). Since the homozygous mutant (AA) genotype was not observed in the study groups for the homozygous and recessive models, risk analysis could not be performed for these models (N/A). The Armitage trend test confirmed a statistically significant trend between variant genotypes and prostate cancer risk (*X* ^2^ = 5.8, *p* = 0.016; Table 3).

#### 3.1.3 rs577451694 Variation

The location of the rs577451694 single nucleotide polymorphism (SNP) in exon 34 of the NOTCH1 gene (NM_017617.5) was determined by NCBI BLAST alignment analysis. In this alignment, the query sequence and the reference genomic region are presented along with neighboring SNPs obtained from the dbSNP database (Material S3).

A statistically significant relationship was found between the distribution of genotype frequencies of the Notch1 rs577451694 variant among the groups (*X* ^2^ = 14.946, *p* = 0.0207). While the CC genotype was not observed at all in the control group (0%), this rate was determined to be 28% (*n* = 7) in the BPH group, 20% (*n* = 5) in the LPCa group, and 52% (*n* = 13) in the MPCa group. The wild‐type homozygous GG genotype was observed at a rate of 93.3% (*n* = 14) in the control group, while it was determined to be 48% in the MPCa group. The heterozygous GC genotype was observed at low frequencies (0%—8%) in all groups (Table 2).

Genotype distributions for the rs577451694 variant showed significant deviations from Hardy–Weinberg equilibrium (HWE) in both the non‐cancerous control group (*p* < 0.001) and the prostate cancer group (*p* < 0.001). When allele frequencies were evaluated, the C allele was found to be statistically significantly higher in the prostate cancer group (38.0%) compared to the non‐cancerous control group (20.0%) (*p* = 0.0012; OR = 2.45, 95% CI: 1.24–4.83). However, no statistically significant difference was found between the two groups in terms of overall genotype distributions (GG, GC, CC) (*X* ^2^ = 3.79, *p* = 0.15; Table 3).

When OR were examined in genetic models based on the C risk allele, it was observed that the CC genotype was significantly associated with prostate cancer risk in the recessive model (OR = 2.65, 95% CI: 1.02–6.89, *p* = 0.04). In the homozygous model, the relationship between the CC genotype and prostate cancer was found to be at the limit of statistical significance (OR = 2.66, 95% CI: 1.00–7.07, *p* = 0.05). No statistically significant increase in risk was observed in the heterozygous and dominant models (*p* = 0.97 and *p* = 0.09, respectively). Finally, the Armitage trend test (Common OR) revealed a statistically highly significant trend between genotypes and prostate cancer risk (*X* ^2^ = 11.2, *p* = 0.0008; Table 3).

### 3.2 Notch3 Gene Variations

#### 3.2.1 rs746192089 Variation

As a result of BLAST alignment performed within the scope of bioinformatics analysis, the rs746192089 variant of the NOTCH3 gene was identified in exon 33. The obtained NCBI BLAST alignment output clearly shows the location of the query sequence mapped to the reference genomic region, as well as the location of neighboring SNPs obtained from the dbSNP database (Material S4).

A statistically significant relationship was found between the groups in terms of genotype frequency distributions of the Notch3 rs746192089 variant (*X* ^2^ = 31.891, *p* < 0.0001).

The homozygous CC genotype was determined as 93.3% (*n* = 14) in the control group, 44% in the BPH group, 28% in the LPCa group, and 8% (*n* = 2) in the MPCa group. In parallel, the heterozygous GC genotype frequency was 6.7% in the control group, while it increased in the BPH (32%), LPCa (52%), and MPCa (68%) groups, respectively. The mutant homozygous GG genotype was not observed at all in the control group, while it showed a similar distribution in the other three groups (20%—24%; Table 2).

Genotype distributions for the rs746192089 variant were found to be consistent with Hardy–Weinberg equilibrium (HWE) in both the non‐cancerous control group (*p* = 0.06) and the prostate cancer group (*p* = 0.20). When allele frequencies were compared, the G allele was found to be significantly higher in the prostate cancer group (52.0%) than in the non‐cancerous group (26.2%) (*p* = 0.0005; OR = 3.04, 95% CI: 1.60–5.77). Similarly, a statistically significant difference was found between the two groups in terms of overall genotype distributions (CC, CG, GG) (*X* ^2^ = 19.45, *p* < 0.001; Table 4).

**TABLE 4:** Genotype and allele frequencies of Notch3 rs746192089, rs1044009 variations in the prostate cancer (LPCa and MPCa) group and non‐cancerous (BPH and Control) group.

SNP rs746192089	Allele		Statistic *p*	OR (95% CI)	Genotype			Statistic *p*	HWE
	C *n* (%)	G *n* (%)			CC	CG	GG		
Non‐cancerous	59 (73.8)	21 (26.2)	**0.0005**	3.04 (1.60–5.77)	25	9	6	***X*** ^*2*^ **= 19.45** ***p* < 0.001**	0.06
Prostate cancer	48 (48.0)	52 (52.0)			9	30	11		0.20
OR (95% CI) (Risk allele G)									
Heterozygous CC vs. CG		Homozygous CC vs. GG		Dominant CC vs. CG + GG		Recessive CC + CG vs. GG		Armitage's trend test	
9.26 (3.10–27.6) ***p* < 0.001**		5.09 (1.36–19.1) ***p* = 0.01**		7.59 (2.76–20.08) ***p* < 0.001**		1.60 (0.51–5.00) *p* = 0.40		***X*** ^*2*^ **= 13.8** ***p*** **= 0.001**	

SNP rs1044009	Allele		Statistic *p*	OR (95% CI)	Genotype			Statistic *p*	HWE
	C *n* (%)	T *n* (%)			CC	CT	TT		
Non‐cancerous	21 (26.2)	59 (73.8)	0.63	1.15 (0.62–2.17)	3	15	22	*X* ^*2*^ = 1.22 *p* = 0.54	0.95
Prostate cancer	29 (29.0)	71 (71.0)			7	15	28		0.50
OR (95% CI) (Risk allele C)									
Heterozygous TT vs. TC		Homozygous TT vs. CC		Dominant TT vs. CT + CC		Recessive TT + TC vs. CC		Armitage's trend test	
0.79 (0.32–1.94) *p* = 0.60		1.83 (0.42–7.93) *p* = 0.41		0.96 (0.41–2.23) *p* = 0.93		2.01 (0.48–8.30) *p* = 0.33		*X* ^*2*^ = 0.52 *p* = 0.47	

*Note:* Bold values indicate statistically significant.

Genetic modeling based on the G risk allele revealed a statistically significant association between the CG genotype and prostate cancer risk in the heterozygous model (OR = 9.26, 95% CI: 3.10–27.6, *p* < 0.001). In the dominant model, carrying either the CG or GG genotype significantly increased cancer risk (OR = 7.59, 95% CI: 2.76–20.08, *p* < 0.001). In the homozygous model, the GG genotype showed a significant association with prostate cancer (OR = 5.09, 95% CI: 1.36–19.1, *p* = 0.01). No statistically significant increase in risk was observed in the recessive model (OR = 1.60, 95% CI: 0.51–5.00, *p* = 0.40; Table 4).

#### 3.2.2 rs1044009 Variation

The location of the rs1044009 single nucleotide polymorphism in exon 33 of the NOTCH3 gene (NM_000435.3) was determined using NCBI BLAST alignment analysis. In this alignment, the query sequence matched with the reference genomic region is displayed along with neighboring SNPs obtained from the dbSNP database (Material S5).

When the genotype frequency distributions of the Notch3 rs1044009 variant were examined, no statistically significant relationship was found between the groups (*X* ^2^ = 3.536, *p* = 0.7392).

The homozygous TT genotype was observed as the most common genotype in all study groups (66.7% in the control group, 48% in the BPH group, 60% in the LPCa group, and 52% in the MPCa group). The heterozygous TC genotype showed a balanced distribution throughout the cohort and was observed between 28% and 40% in the groups. The CC genotype was not observed at all in the control group; however, it was detected at very similar frequencies in the BPH (12%), LPCa (12%), and MPCa (16%) groups (*p* > 0.05; Table 2).

Genotype distributions for the rs1044009 variant were found to be consistent with Hardy–Weinberg equilibrium (HWE) in both the non‐cancerous control group (*p* = 0.95) and the prostate cancer group (*p* = 0.50). When allele frequencies were compared, no statistically significant difference was found between the prostate cancer group and the non‐cancerous control group in terms of the distribution of C and T alleles (*p* = 0.63; OR = 1.15, 95% CI: 0.62–2.17). In parallel, no significant difference was found between the two groups in terms of overall genotype distributions (CC, CT, TT) (*X* ^2^ = 1.22, *p* = 0.54; Table 4).

When OR was evaluated in genetic models based on the C risk allele, no statistically significant association was found between the heterozygous (*p* = 0.60), homozygous (*p* = 0.41), dominant (*p* = 0.93), and recessive (*p* = 0.33) models in terms of prostate cancer risk. Furthermore, the Armitage trend test confirmed that there was no significant trend between variant genotypes and prostate cancer risk (*X* ^2^ = 0.52, *p* = 0.47; Table 4).

### 3.3 Protein Levels

#### 3.3.1 Notch1 Protein Levels

When Notch1 protein levels were examined in the study groups, the mean protein level in the control group was determined as 77.21 ± 19.76. In the benign prostatic hyperplasia (BPH) group, Notch1 protein levels were 51.91 ± 10.24; in the localized prostate cancer (PCa) group 30.63 ± 9.53; and in the metastatic prostate cancer (MPCa) group 26.22 ± 3.11.

In intergroup comparisons, no statistically significant difference was found between the control and BPH groups in terms of Notch1 protein levels (*p* = 0.076). However, statistically significant differences were found between the control group and the localized prostate cancer group (*p* = 0.006) and between the control group and the metastatic prostate cancer group (*p* = 0.003) in terms of Notch1 protein levels. These findings indicate that Notch1 protein levels are lower, particularly in the localized prostate cancer and metastatic prostate cancer groups, compared to the control group (Figure 1).

**FIGURE 1:**
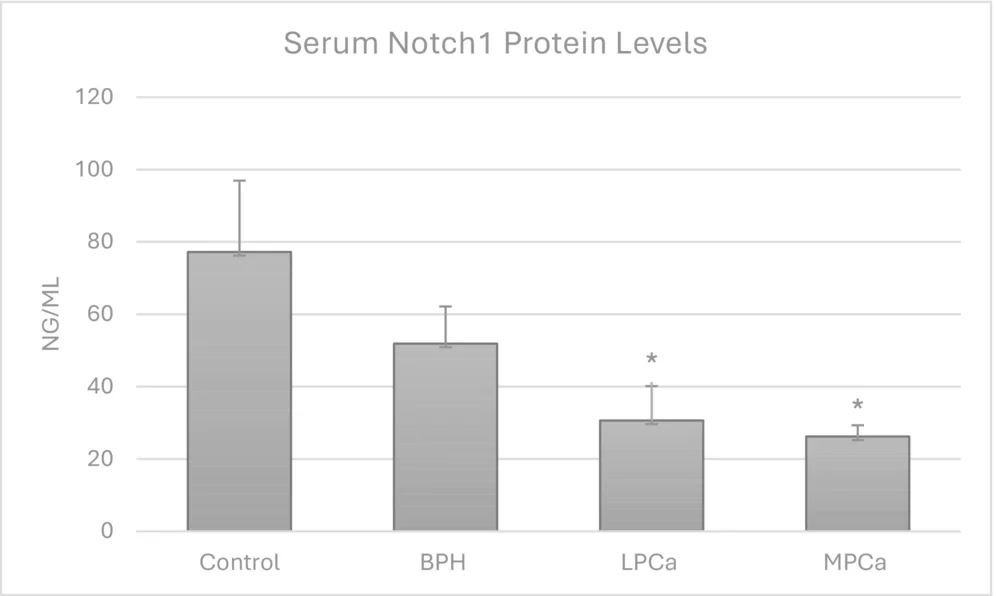
Comparison of serum NOTCH1 levels between the patient and control groupsMann–Whitney rank sum test, Control‐BPH: There was no statistically significant difference (*p* = 0.076); Control‐MPCa: There was a statistically significant s (*p* = 0.003); Control‐LPCa: There was a statistically significant difference (*p* = 0.006), * *p* < 0.05.

When protein levels were compared with genotypes, a statistically significant difference was found in Notch1 protein levels among 3 different genotype groups of the Position 54,305 variation (*p* = 0.036). In the pairwise comparison (Post hoc Dunn/Bonferroni) analysis performed to determine which groups caused the difference, it was determined that the significant difference was between the TA and TT genotypes (adjusted *p* = 0.033). Individuals with the TT genotype had significantly higher Notch1 protein levels than those with the TA genotype. No significant difference was found between the AA genotype and the other groups. When the rs2133314984 variation was evaluated, it was determined that the majority of the samples had the TT genotype. No statistically significant difference was found in Notch1 protein levels between the TA (*N* = 11) and TT (*N* = 79) genotypes (*p* = 0.395). Notch1 protein levels were found to be statistically similar among CC, GC, and GG genotypes with the rs577451694 variation; no significant difference was observed between the groups (*p* = 0.245; Table 5).

**TABLE 5:** Comparative analysis of NOTCH1 and NOTCH3 genotype distributions in study groups with serum protein levels.

Notch1 variations	Genotype	*N*	Mean ± standard deviation	*p*
Position 54,305	AA	25	43.77 ± 48.66	**0.036**
	TA	38	29.98 ± 22.95	
	TT	27	74.95 ± 73.37	
rs2133314984	TA	11	33.80 ± 27.77	0.395
	TT	79	49.18 ± 55.31	
rs577451694	CC	25	27.80 ± 21.74	0.245
	GC	4	53.33 ± 41.98	
	GG	61	54.90 ± 60.39	

Notch3 variations	Genotype	*N*	Mean ± standard deviation	*p*
rs746192089	CC	34	13.20 ± 15.83	**< 0.001**
	GC	39	3.81 ± 9.40	
	GG	17	5.80 ± 8.49	
rs1044009	TT	50	7.60 ± 13.05	0.937
	TC	30	8.32 ± 13.24	
	CC	10	6.68 ± 10.56	

*Note:* Bold values indicate statistically significant.

#### 3.3.2 Notch3 Protein Levels

When Notch3 protein levels were evaluated in the study groups, the mean protein level in the control group was determined as 22.39 ± 4.86. In the benign prostatic hyperplasia group, the Notch3 protein level was found to be 6.52 ± 1.51, in the localized prostate cancer group 6.70 ± 2.41, and in the metastatic prostate cancer group 1.19 ± 0.69. In intergroup comparisons, statistically significant differences were found in Notch3 protein levels between the control group and the benign prostatic hyperplasia group (*p* = 0.002), between the control group and the localized prostate cancer group (*p* < 0.001), and between the control group and the metastatic prostate cancer group (*p* < 0.001). In addition, the lowest Notch3 protein level was determined to be in the metastatic prostate cancer group (Figure 2).

**FIGURE 2:**
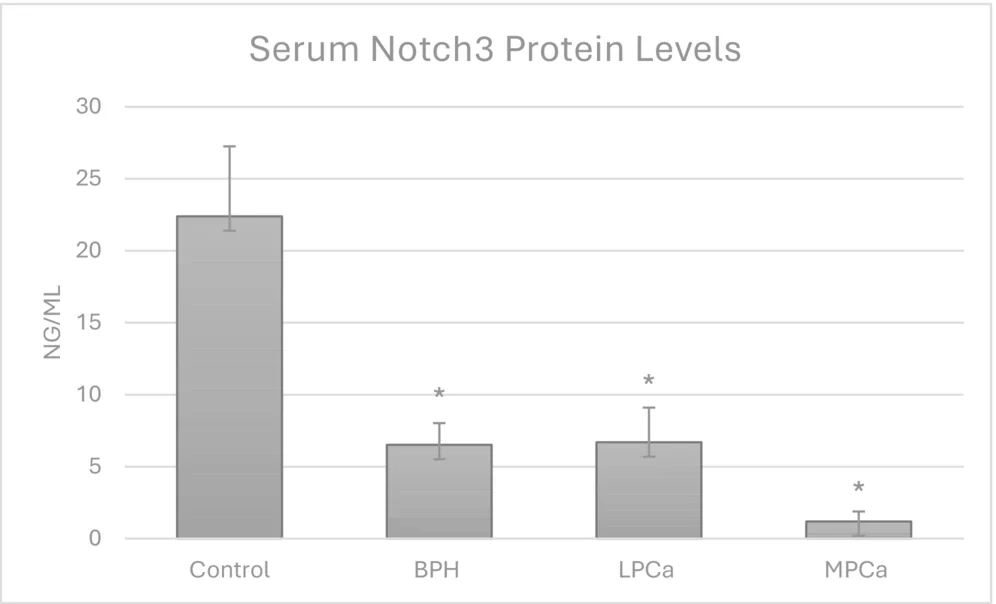
Comparison of serum NOTCH3 levels between the patient and control groups. Mann–Whitney rank sum test, Control‐BPH: There was a statistically significant difference (*p* = 0.002); Control‐MPCa: There was a statistically significant difference (*p* < 0.001); Control‐LPCa: There was a statistically significant difference (*p* < 0.001), *p < 0.05.

When comparing the effects of rs746192089 and rs1044009 variations on Notch3 protein levels, a significant difference was found in Notch3 protein levels among genotypes with respect to the rs74619208 variation (*p* < 0.001). In post hoc evaluations, individuals with the CC genotype (13.20 ± 15.83) were observed to have higher Notch3 protein levels compared to individuals with the GC and GG genotypes. Additionally, no statistically significant difference was found in Notch3 protein levels among TT, TC, and CC genotypes for the rs1044009 variation (*p* = 0.937; Table 5).

## 4 Discussion

Prostate cancer represents one of the most prevalent malignancies among men and is profoundly dependent on androgen receptor (AR) signaling during its initial stages. Consequently, the cornerstone of therapeutic intervention has long been established as androgen deprivation therapy (ADT), aimed at the suppression of the AR pathway. However, a significant proportion of tumor cells eventually develop resistance to this treatment, progressing into the castration‐resistant prostate cancer (CRPC) phenotype, which is characterized by increased aggressiveness and poor clinical prognosis. This progression underscores the critical necessity of elucidating the alternative molecular mechanisms and signaling pathways involved in prostate cancer advancement. In particular, the cross‐talk between the AR and Notch signaling pathways is considered to play a pivotal role in the tumor's escape strategies from hormonal suppression [12, 13].

The Notch signaling pathway has been demonstrated to regulate the tumorigenic capacity of prostate cancer stem cells, while modulating oncogenic proliferation and chemoresistance. Furthermore, activation of this pathway has been reported to trigger metastatic progression in prostate cancer cells by facilitating increased migration, invasion, and epithelial‐to‐mesenchymal transition (EMT) [14].

Among the Notch receptors, NOTCH1 has been specifically shown to be highly expressed in malignant prostate cells, with its active nuclear form (NICD1) demonstrating significant upregulation. Furthermore, it has been established that the loss of this receptor can lead to a reduction in tumorigenesis and metastatic potential [15].

However, the activation mechanisms of the Notch pathway in prostate cancer have not yet been fully elucidated, and there is a growing body of research aimed at revealing the contributions of genetic and molecular alterations within this pathway to disease development and progression. In the present study, variations in the NOTCH1 and NOTCH3 gene regions, along with serum protein levels, were evaluated collectively across prostate pathologies (BPH and Prostate Cancer), and our findings are discussed in this section.

### 4.1 NOTCH1 Gene Position 54,305 Variation

Following sequence analysis, the g.54305 T>A variation located in exon 34 of the *NOTCH1* gene was identified as a novel nucleotide substitution that is not listed in the dbSNP database and has not been previously reported in the literature.

It is well established that the proteasomal degradation of the NOTCH1 protein is regulated via the PEST domain at the C‐terminus, which provides a critical binding surface for the E3 ubiquitin ligase FBXW7. In the existing literature, Exon 34, which encodes this domain, is recognized as a mutational hotspot where alterations cluster to sustain oncogenic activity by preventing signal termination [16, 17]. The localization of the novel g.54305 T>A variation within this critical functional region suggests a potential impact on protein stability.

In this study, genotype comparisons in Control, BPH, LPCa, and MPCa groups revealed significantly higher carrier status (TA and AA) of the mutant A allele at Notch1 Position 54,305 locus in the benign prostatic hyperplasia and prostate cancer groups compared to the control group. This suggests that this variation may play a triggering role in proliferative processes in prostate tissue. The decrease in the dominant wild‐type TT genotype in the control group as cancer stage progresses indicates that this genotype may be involved in a protective mechanism against prostate malignancies. Given the critical role of the Notch1 pathway in cell differentiation and the balance between tumor suppression and oncogenic transformation, it can be hypothesized that nucleotide changes at Position 54,305 locus alter the activation level of the pathway, thereby predisposing to tumor development.

When genotypes were evaluated considering prostate cancer (LPCa + MPCa) and non‐cancerous groups (Control+BPH), our analysis revealed a significant association between this variation and prostate cancer. The mutant A allele frequency was found to be significantly higher in the patient group, and genetic modeling showed that the homozygous AA genotype significantly increased the risk of disease. These statistical findings suggest that the g.54305 T>A variation may be a potential genetic risk factor in the development of prostate cancer, possibly by disrupting the PEST domain and inhibiting signal termination. Although our results show an association, further functional studies and validations in larger cohorts are recommended to fully elucidate its role in disease progression.

### 4.2 NOTCH1 Gene rs2133314984 Variation

The rs2133314984 variation examined in this study is a single nucleotide variant (SNV) located at position 136,496,328 (GRCh38) on chromosome 9 within the *NOTCH1* gene. The Adenine to Thymine (A>T) substitution (c.7411T>A) occurring at position 7411 in the coding region (Exon 34) represents a missense mutation, leading to a p.Ser2471Thr amino acid change at the protein level. The rs2133314984 (p.Ser2471Thr) variation is situated within the C‐terminal PEST domain of the NOTCH1 protein, which is responsible for signal termination (https://www.ncbi.nlm.nih.gov/snp/?term=rs2133314984). The observation that the Minor Allele Frequency (MAF) of this variant is below 0.1% in the gnomAD database indicates that it represents a rare genetic alteration (https://gnomad.broadinstitute.org/variant/9‐136496328‐T‐A).

In genotype comparisons performed in the control, BPH, LPCa, and MPCa groups, no statistically significant association was found between the Notch1 rs2133314984 polymorphism and prostate cancer risk or progression. This suggests that variation at this locus is not a direct primary driver in the etiopathogenesis of prostate diseases. Although not statistically significant, it is noteworthy that the heterozygous TA genotype (20%) showed a numerically increasing trend in the LPCa and MPCa groups, in contrast to the TT genotype (100%) in the control group. Further studies in cohorts with larger patient numbers are needed to clarify whether this trend has biological significance.

When genotypes were evaluated considering prostate cancer (LPCa + MPCa) and non‐cancerous groups (Control + BPH), in contrast to its rarity in the general population, our analyses revealed a statistically significant association between the rs2133314984 variation and prostate cancer. The frequency of the mutant A allele was found to be significantly higher in the patient group, and genetic modeling demonstrated that carrying the variant allele (TA genotype) significantly increased the risk of disease. Since this transposition between serine and threonine within the PEST domain has the potential to modulate post‐translational modification processes and disrupt signal termination, these statistical findings suggest a possible active role in prostate cancer pathogenesis. However, to fully confirm these clinical implications, our findings need to be supported by larger population screenings and functional in vitro analyses in the future.

### 4.3 NOTCH1 Gene rs577451694 Variation

The rs577451694 variation is a single nucleotide variant (SNV) located within the 3′ untranslated region (3′ UTR) of the *NOTCH1* gene, corresponding to the genomic position 136,496,047 (GRCh38) on chromosome 9. This variant, which leads to a c.*24G>T/C/A substitution in the mRNA transcript, does not result in any amino acid change within the protein‐coding sequence (https://www.ncbi.nlm.nih.gov/snp/?term=rs577451694).

The 3′ untranslated regions (3′ UTRs) serve as pivotal domains that determine mRNA stability and translational efficiency. Genetic variants, specifically single nucleotide polymorphisms (SNPs) within miRNA binding sites, particularly in the 3′ UTR, can modulate target gene expression by altering miRNA‐mRNA interactions, thereby increasing cancer susceptibility and influencing prognosis. These variants may disrupt existing binding sites or create de novo ones, consequently impacting tumorigenesis across various cancer types [18]. Due to its localization within the 3′ UTR of the NOTCH1 gene, the rs577451694 variation possesses the potential to interact with miRNAs, which are key players in post‐transcriptional regulation. A single nucleotide substitution may disrupt miRNA‐mRNA pairing, thereby altering the intracellular half‐life of NOTCH1 mRNA and, consequently, protein expression levels.

Comparison across all groups revealed that the Notch1 rs577451694 variant was detected at a very high frequency (52%) in the rare homozygous CC genotype metastatic prostate cancer (MPCa) group. This suggests that the CC genotype may be a risk factor in the process of prostate tumors becoming more aggressive and metastatic. The gradual decrease in the wild‐type GG genotype and the increase in the CC genotype as the disease progresses suggest a possible association between genetic alteration at this locus and metastatic mechanisms such as invasive tumor cells and epithelial–mesenchymal transition (EMT).

Evaluation of genotypes based on prostate cancer (LPCa+MPCa) and non‐cancerous groups (Control + BPH) has shown that a statistically significant association between the rs577451694 variation and prostate cancer risk. Specifically, the C allele was found at a significantly higher frequency in the patient group, and genetic modeling showed that the homozygous CC genotype increased the risk of disease.

Given its localization in the 3′ UTR region, these significant findings support the hypothesis that this variation may disrupt miRNA‐mRNA coupling, thereby altering NOTCH1 mRNA stability and contributing to prostate cancer pathogenesis. How this functional alteration affects the course of the disease on an individual basis, particularly through interactions with other genetic and epigenetic factors, awaits further elucidation through comprehensive functional genomics studies.

### 4.4 NOTCH3 Gene rs746192089 Variation

Located at position 15,161,668 (GRCh38) on chromosome 19, rs746192089 is a missense mutation. A cytosine‐to‐thymine (C>T) substitution at position 5960 of the coding sequence results in the replacement of Alanine with Valine at position 1987 at the protein level (p.Ala1987Val) (https://www.ncbi.nlm.nih.gov/snp/?term=rs746192089).

Amino acid substitutions of this nature occurring in the cytoplasmic tail of the NOTCH3 protein (NICD3) can influence protein stability, the rate of nuclear translocation, and consequently, the transcriptional activation of target genes, such as those in the Hes/Hey family [19]. Considering the role of the NOTCH3 pathway in prostate cancer cells, particularly in chemotherapy resistance [20] and the maintenance of the cancer stem cell phenotype [21], the effects of this variant on the oncogenic potential of the pathway are of significant functional importance.

The results of the analyses evaluating all groups and genotypes showed highly significant differences between groups in the genotype distribution of the Notch3 rs746192089 polymorphism. The protective CC genotype, which was 93.3% in the control group, decreased to 8% in the metastatic stage, while the heterozygous GC genotype was determined to be 68% in the MPCa group, indicating that the mutant G allele may play a critical role in disease progression. The gradual increase of the heterozygous (GC) variant from BPH (32%) to localized cancer (52%) and metastatic stage (68%) suggests that this locus may be useful in monitoring prostate diseases and predicting the aggressiveness of the tumor.

When genotypes were evaluated considering prostate cancer (LPCa + MPCa) and non‐cancerous groups (Control + BPH), a statistically significant increase in the frequency of the G allele of the rs746192089 variant within the NOTCH3 gene was observed in the prostate cancer group compared to the non‐cancerous group.

Literature reviews have revealed no studies investigating the association of the rs746192089 variant with prostate cancer or other malignancies; thus, current data suggest that this genetic alteration is being evaluated for the first time in the context of prostatic diseases. Given the statistically significant association found in our analysis, further studies supported by functional analyses are needed to elucidate the role of the rs746192089 variant in prostate cancer pathogenesis and its effect on the Notch signaling pathway.

### 4.5 NOTCH3 Gene rs1044009 Variation

The rs1044009 variant evaluated within the scope of this study is a missense mutation located at position 15,160,960 (GRCh38) on chromosome 19 and is described in the literature as a common polymorphism. A C>T substitution at position 6668 of the coding region results in a p.Ala2223Val amino acid substitution at the protein level. Classified as benign in the clinical database (ClinVar), this variant possesses a notably high minor allele frequency (MAF > 0.20) across various populations (https://www.ncbi.nlm.nih.gov/snp/?term=rs1044009).

Although it is considered not to cause a major functional disruption in the protein structure, the literature discusses the combinatorial effects of such common polymorphisms with other rare variants, as well as the modifying roles they may play as part of the genetic background in complex diseases such as prostate cancer.

Although the NOTCH3 rs1044009 variant has been investigated in the literature in relation to various cancer types, it is reported that the effects of this polymorphism on tumor biology may vary depending on the cancer type and clinical context. In a study investigating variations in genes associated with tumor dormancy, the NOTCH3 rs1044009 variation was shown to be associated with survival in patients with colorectal liver metastasis receiving neoadjuvant bevacizumab‐based chemotherapy. The study reported that this polymorphism was significantly associated with 3‐year overall survival rates, and this association remained significant in multivariate analysis. Furthermore, it was stated that the same variation could be one of the effective SNPs in predicting treatment response and clinical outcomes [22, 23].

In other studies, investigating the association between genetic variations in the Notch signaling pathway and cancer development, it has been demonstrated that NOTCH3 polymorphisms may be associated with tumor progression and clinical characteristics. In a study conducted on colorectal cancer patients, it was reported that polymorphisms in the NOTCH3 gene showed a significant association with advanced TNM stage, and specific genotypes were linked to increased protein expression. The same study stated that individuals carrying the Notch3 variant allele may have a higher risk of colorectal cancer and that survival rates in these patients were lower. These findings suggest that Notch receptor polymorphisms, particularly for *NOTCH3*, may serve as potential biomarkers in terms of cancer development and prognosis [24].

Similarly, genetic studies on lung cancer have demonstrated that certain polymorphisms of the NOTCH3 gene are associated with disease risk. In a study conducted within the Turkish population, it was reported that the NOTCH3 rs3815188 and rs1043994 polymorphisms showed significant differences in genotype and allele frequencies between lung cancer patients and control groups, suggesting that these variants may be associated with genetic susceptibility to the disease [25].

While these findings, when considered collectively, suggest that polymorphisms in the NOTCH3 gene may be associated with tumor development, progression, and clinical outcomes in various cancer types, our study revealed that both the genotype comparison of all groups and the comparison of cancerous/non‐cancerous groups showed a similar distribution of genotype frequencies of the Notch3 rs1044009 variation in both benign and malignant groups compared to the control group; this suggests that this variant has no effect on prostate cancer susceptibility or metastatic processes.

### 4.6 Evaluation of Notch1 and Notch3 Protein Levels

In addition to genetic variation analyses, protein level assessments also yielded significant results. Serum NOTCH1 protein levels were found to be significantly lower in the prostate cancer and metastatic prostate cancer groups compared to the control group. Similarly, NOTCH3 protein levels were significantly reduced in the BPH, prostate cancer, and especially metastatic prostate cancer groups compared to the control group, with the lowest levels detected in the metastatic prostate cancer group. These findings demonstrate that the genetic variations examined show a statistically significant association with disease risk, as well as changes in Notch pathway protein levels that may be associated with disease progression. Our results suggest that the role of the Notch signaling pathway in prostate diseases is not limited to activation alone, but may exhibit different regulatory patterns depending on the context.

Abnormal Notch signaling contributes to several key features of cancer and is particularly closely associated with the emergence of highly aggressive tumors characterized by poor prognosis [26]. The Notch pathway regulates the tumorigenic capacity of prostate cancer stem cells, modulates oncogenic proliferation and chemotherapy resistance; furthermore, it has been reported that activation of this pathway may be associated with aggressive tumor features such as increased migration, invasion, and epithelial‐mesenchymal transition (EMT) in prostate cancer cells [27].

Among Notch receptors, NOTCH1 is particularly highly expressed in malignant prostate cells, and loss of this receptor has been shown to reduce tumor formation and metastatic potential [15]. However, it is known that members of the Notch family are frequently dysregulated in various cancer types, and these cellular changes can lead to both oncogenesis and tumor suppression, depending on the context. Indeed, it has been reported that in oral squamous cell carcinoma (OSCC), NOTCH1, NOTCH2, and NOTCH4 are prominently expressed, while NOTCH3 expression is not detected, and high methylation levels are found in the NOTCH3 promoter region. Moreover, clinical samples have shown that NOTCH3 methylation is significantly higher in tumor tissues compared to normal tissues [28].

Similarly, there is evidence that the Notch signaling pathway may exhibit tumor suppressor properties in various cancer types. For example, in breast cancer, NOTCH3 has been reported to inhibit metastasis by transcriptionally stimulating STAT5A through mechanisms associated with immune cells infiltrating the tumor [29]. Furthermore, in small cell lung cancer (SCLC), NOTCH1 signaling has been shown to increase cellular apoptosis, decrease proliferative capacity, and inhibit epithelial‐mesenchymal transition, thereby reducing cell motility, invasion, and metastatic potential. It has also been reported to exhibit a general tumor suppressor effect, modulating chemotherapy resistance by influencing multidrug resistance‐associated protein‐1 (MRP‐1) levels. It is suggested that inducing NOTCH1 using histone deacetylase inhibitors (HDACs) for SCLC could be a potential therapeutic approach. However, it is emphasized that the effect of such a complex signaling pathway on tumor carcinogenesis cannot be generalized to all human cancers, and its effects may vary depending on the cellular context [30].

Recent studies have shown that expression levels of NOTCH family genes can also show significant changes in prostate cancer. It has been reported that the transcription levels of NOTCH1 and NOTCH4 are significantly lower in prostate cancer tissues compared to normal tissues. These results suggest that suppression of NOTCH family genes in prostate cancer tissues may be associated with tumorigenesis [31].

One of the key findings in this study is the gradual decrease in serum Notch1 and Notch3 levels observed as the clinical stages of prostate cancer progress. This result differs from some studies reporting increased Notch expression in localized prostate tumor tissues. A possible explanation for this is that there may not be a direct linearity between the expression levels of Notch receptors in the tissue and their detectable amounts in circulation. Changes in the ectodomain shedding or proteolytic processing of Notch proteins, which are essentially transmembrane receptors, may directly affect the amount of soluble Notch released into the blood. Accordingly, it has been reported that differences in the activity of regulatory enzymes, including ADAM proteases involved in Notch processing, may contribute to this discrepancy between tissue and circulating Notch levels in various malignancies [32].

The fact that circulating Notch proteins do not originate from a single source is another factor that complicates the interpretation of serum measurements. Notch signaling is likely to originate from multiple cellular foci, including tumor cells as well as stromal, endothelial, and immune cell populations in the tumor microenvironment. Notch signaling has been shown to be involved in many steps in the regulation of the tumor microenvironment, including immune cells, stromal compartments, and angiogenic processes [33, 34]. Therefore, although we cannot definitively elucidate the underlying mechanisms with our current data, the low serum levels we detected in our cohort may be a reflection of systemic biological changes associated with disease progression.

The interpretation of these findings is further complicated by the known context‐dependent functions of the Notch signaling pathway in prostate cancer. Notch signaling is known to exert tumor‐promoting or tumor‐suppressive effects depending on the cellular and molecular environment in which it is located [35]. Consequently, the gradual decrease in serum Notch1 and Notch3 levels observed in this study should be approached with caution; further mechanistic studies are needed to clarify the cellular origins, biological significance, and potential clinical value of these findings.

Other findings from our study show a correlation between NOTCH1 gene variations and serum Notch1 protein levels. Specifically, a statistically significant relationship was found between the genotype distribution at position 54,305 and serum protein concentrations. Individuals with the homozygous mutant or the TT genotype, described as rare/specific in the literature, showed higher serum protein levels compared to homozygous AA genotypes, which have a strong association with heterozygous TA and disease risk. This suggests that this nucleotide change may have an upregulatory effect on protein expression mechanisms, mRNA stabilization, or post‐translational modification processes of the protein.

No statistically significant difference was found between serum NOTCH1 levels and the other two variations examined, rs2133314984 and rs577451694 genotypes. This finding indicates that not every polymorphism in the regulation of the Notch1 signaling pathway is reflected equally in expression levels, and that the functional effects of the variation at position 54,305, in particular, need to be evaluated in more detail.

Analyses performed with NOTCH3 variant genotypes revealed a statistically significant association between the rs746192089 polymorphism and serum Notch3 protein levels. When genotypes were examined, it was determined that individuals with the homozygous CC genotype had significantly higher average serum protein levels than those with the GG genotype. This decrease in serum protein levels observed in the presence of the G variant (both heterozygous and homozygous mutant cases) suggests that the rs746192089 polymorphism may play a repressive role in the Notch3 signaling system or lead to a conformational change that accelerates protein degradation. In contrast, no significant difference was found between the rs1044009 variation and protein levels. Therefore, further analyses are anticipated to confirm the effect of the rs746192089 polymorphism on the Notch3 expression profile.

Our findings reveal that the genetic variations examined significantly increase the risk of prostate cancer; conversely, there is a marked decrease in NOTCH1 and NOTCH3 protein levels, particularly in the prostate cancer and metastatic prostate cancer groups. The lowest NOTCH3 protein levels were detected in the metastatic prostate cancer group, suggesting that the Notch signaling pathway may undergo more pronounced changes in the later stages of the disease.

## 5 Conclusion

In conclusion, this study evaluated NOTCH1 and NOTCH3 gene variations and serum protein levels in patients with BPH, LPCa, and MPCa. Our analyses showed that specific NOTCH1 and NOTCH3 variants were statistically significantly associated with an increased risk of prostate cancer. In addition to these genetic findings, a significant decrease in NOTCH1 and NOTCH3 protein levels was observed, particularly in the LPCa and MPCa groups. These findings suggest that alterations in the Notch signaling pathway may play a role in prostate cancer pathogenesis and progression, and that members of the NOTCH family may assume context‐dependent roles in prostate tumor biology. However, further research involving larger patient cohorts and supported by functional studies is needed to better understand the biological significance of these results and to evaluate the Notch pathway as a potential biomarker or therapeutic target.

## 6 Limitations

The limited sample size in our study may have restricted the ability to statistically demonstrate potential associations between the investigated SNP variants and the disease groups. While the translational generalization of serum Notch1 and Notch3 as definitive biomarkers warrants future validation in larger, multi‐center, and longitudinal patient cohorts, the statistical reliability of the current study design is supported by post hoc analysis. To address potential considerations regarding the sample size, a post hoc power analysis was conducted based on the observed primary endpoints. The analysis indicated an effect size (Cohen's *f*) of 1.672 for serum Notch1 and 2.786 for serum NOTCH3, both corresponding to a statistical power of 100% (1−*β* = 1.00). These metrics suggest that the current cohort (*N* = 90) provides adequate statistical proficiency to detect the observed differences among the clinical groups, aligning well with the conventional 80% statistical power threshold. Nevertheless, further validation through highly stratified prospective studies remains essential to more precisely evaluate potential diagnostic cut‐off thresholds and long‐term predictive utility across diverse patient populations. However, a limitation of the present study is the small sample size in clinical subgroups in terms of genetic association analyses. While the cohort size (*n* = 90) is statistically sufficient to assess variations in circulating serum profiles, it lacks the necessary statistical power to establish definitive genotypic associations, particularly for single nucleotide polymorphisms (SNPs) with low minor allele frequencies. This statistical limitation is reflected in the wide confidence intervals and unstable odds ratios observed for specific variants. Consequently, the genetic findings presented here should be considered exploratory, serving as a preliminary screening requiring further validation in larger, robust multicenter genotypic cohorts. Furthermore, only specific regions of the NOTCH1 and NOTCH3 genes were analyzed, and potential genetic alterations in other components of the Notch signaling pathway were not evaluated. Additionally, functional analyses aimed at elucidating the biological effects of the identified variations or the observed changes in protein levels were not performed. Finally, as the study was conducted within a single‐center patient cohort, further studies in larger and independent populations are required to verify the generalizability of the findings.

## Author Contributions

E.Y. and C.O. designed the study and supervised the project. Data collection was performed by A.O. and E.Y. Methodology and experimental procedures were carried out by E.Y., C.O., and G.Z. Formal analysis and investigation were conducted by E.Y. and C.O. The original manuscript was drafted by E.Y. and C.O., and subsequently reviewed and edited by E.Y., C.O., and H.K. Funding for the research was acquired by C.O. All authors have reviewed and approved the final version of the manuscript.

## Funding

This work was supported by a research grant funded by the Research Foundation of Kutahya Health Sciences University, Kutahya, Turkey (Ref. No: TSA‐2021‐65).

## Ethics Statement

The study was approved by the Eskişehir Osmangazi University Clinical Research Ethics Committee decisions numbered 2020/31.

## Consent

All individuals provided informed consent in the study in accordance with the principles of the Declaration of Helsinki.

## Conflicts of Interest

The authors declare no conflicts of interest.

## Supporting information


**Material S1:** NCBI BLAST alignment of *NOTCH1* exon 34 sequence (NM_017617.5) showing the novel g.54305 T>A variation absent in the dbSNP (v157) database.
**Material S2:** NCBI BLAST alignment of *NOTCH1* exon 34 showing the genomic localization of the rs2133314984 variant and neighboring dbSNP variants.
**Material S3:** NCBI BLAST alignment of *NOTCH1* exon 34 (NM_017617.5) showing the genomic localization of the rs577451694 SNP and neighboring dbSNP variants.
**Material S4:** NCBI BLAST alignment of *NOTCH3* exon 33 showing the genomic localization of the rs746192089 variant and neighboring dbSNP variants.
**Material S5:** NCBI BLAST alignment of *NOTCH3* exon 33 (NM_000435.3) showing the genomic localization of the rs1044009 SNP and neighboring dbSNP variants.

## Data Availability

The datasets used or analysed during the current study are available from the corresponding author on reasonable request. Clinical trial number: not applicable.
