# Supplementary material for: Analysis of Notch1 and Notch3 Signaling Pathway Components in Benign Prostatic Hyperplasia and Prostate Cancer Patients
Source: J Clin Lab Anal. 2026 Aug 18;40(19):e70339. doi: 10.1002/jcla.70339 (PMC13483564; doi:10.1002/jcla.70339)
Supplement: Supplementary file 1 [file JCLA-40-e70339-s001.zip › jcla70339-sup-0001-Supinfo1@Supplementary caption1.docx]

**Supplementary Material 1.** NCBI BLAST alignment of *NOTCH1* exon 34 sequence (NM_017617.5) showing the novel g.54305 T>A variation absent in the dbSNP (v157) database.
