# Supplementary material for: Analysis of Notch1 and Notch3 Signaling Pathway Components in Benign Prostatic Hyperplasia and Prostate Cancer Patients
Source: J Clin Lab Anal. 2026 Aug 18;40(19):e70339. doi: 10.1002/jcla.70339 (PMC13483564; doi:10.1002/jcla.70339)
Supplement: Supplementary file 1 [file JCLA-40-e70339-s001.zip › jcla70339-sup-0004-Supinfo4@Supplementary caption4.docx]

**Supplementary Material 4.** NCBI BLAST alignment of *NOTCH3* exon 33 showing the genomic localization of the rs746192089 variant and neighboring dbSNP variants.
