# Supplementary material for: Analysis of Notch1 and Notch3 Signaling Pathway Components in Benign Prostatic Hyperplasia and Prostate Cancer Patients
Source: J Clin Lab Anal. 2026 Aug 18;40(19):e70339. doi: 10.1002/jcla.70339 (PMC13483564; doi:10.1002/jcla.70339)
Supplement: Supplementary file 1 [file JCLA-40-e70339-s001.zip › jcla70339-sup-0005-Supinfo5@Supplementary caption5.docx]

**Supplementary Material 5.** NCBI BLAST alignment of *NOTCH3* exon 33 (NM_000435.3) showing the genomic localization of the rs1044009 SNP and neighboring dbSNP variants.
