# Supplementary material for: Analysis of Notch1 and Notch3 Signaling Pathway Components in Benign Prostatic Hyperplasia and Prostate Cancer Patients
Source: J Clin Lab Anal. 2026 Aug 18;40(19):e70339. doi: 10.1002/jcla.70339 (PMC13483564; doi:10.1002/jcla.70339)
Supplement: Supplementary file 1 [file JCLA-40-e70339-s001.zip › jcla70339-sup-0006-Supinfo6@Supplementary Material1_Position 54305.pdf]

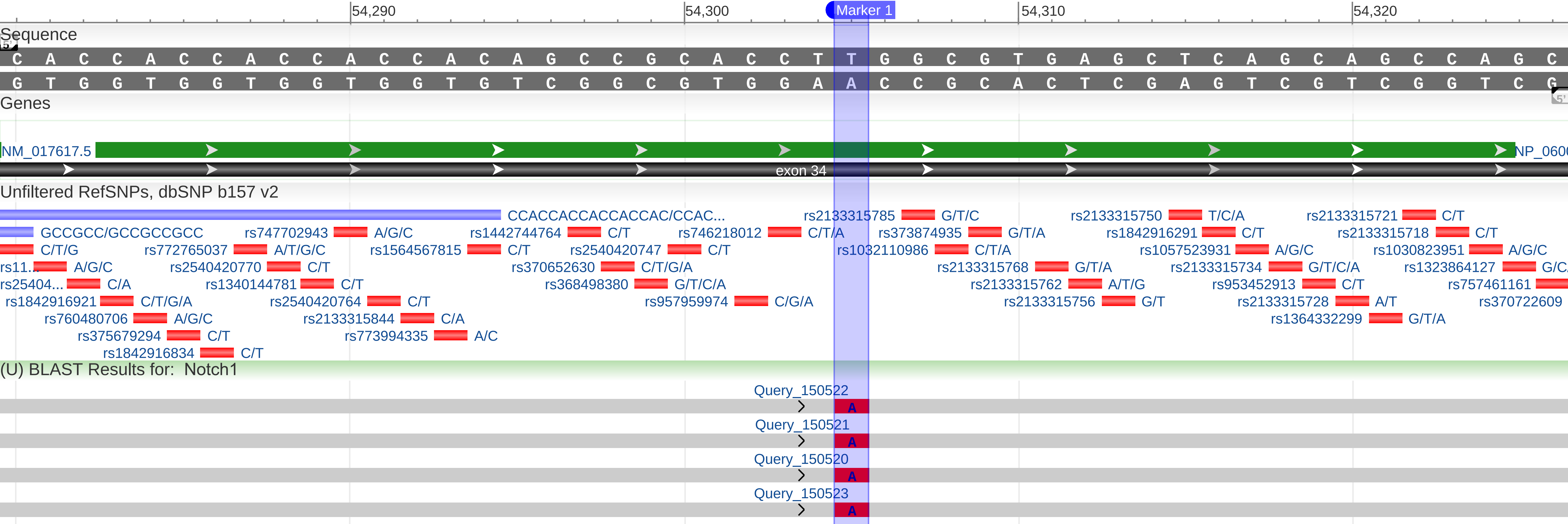

54,290
54,300
Marker 1
54,310
54,320
Sequence
C A C C A C C A C C A C C A C A G C C G C A C C T T G G C G T G A G C T C A G C A G C C A G C
G T G G T G G T G G T G G T G T C G G C G T G G A A C C G C A C T C G A G T C G T C G G T C G
Genes
NM_017617.5
NP_0600
exon 34
Unfiltered RefSNPs, dbSNP b157 v2
CCACCACCACCACCAC/CCAC...
rs2133315785 G/T/C
rs2133315750 T/C/A
rs2133315721 C/T
GCCGCC/GCCGCCGCC
rs747702943 A/G/C
rs1442744764 C/T
rs746218012 C/T/A
rs373874935 G/T/A
rs1842916291 C/T
rs2133315718 C/T
C/T/G
rs772765037 A/T/G/C
rs1564567815 C/T
rs2540420747 C/T
rs1032110986 C/T/A
rs1057523931 A/G/C
rs1030823951 A/G/C
rs11... A/G/C
rs2540420770 C/T
rs370652630 C/T/G/A
rs2133315768 G/T/A
rs2133315734 G/T/C/A
rs1323864127 G/C
rs25404... C/A
rs1340144781 C/T
rs368498380 G/T/C/A
rs2133315762 A/T/G
rs953452913 C/T
rs757461161
rs1842916921 C/T/G/A
rs2540420764 C/T
rs957959974 C/G/A
rs2133315756 G/T
rs2133315728 A/T
rs370722609
rs760480706 A/G/C
rs2133315844 C/A
rs1364332299 G/T/A
rs375679294 C/T
rs773994335 A/C
rs1842916834 C/T
(U) BLAST Results for: Notch1
Query_150522
A
Query_150521
A
Query_150520
A
Query_150523
A
