# Supplementary material for: Analysis of Notch1 and Notch3 Signaling Pathway Components in Benign Prostatic Hyperplasia and Prostate Cancer Patients
Source: J Clin Lab Anal. 2026 Aug 18;40(19):e70339. doi: 10.1002/jcla.70339 (PMC13483564; doi:10.1002/jcla.70339)
Supplement: Supplementary file 1 [file JCLA-40-e70339-s001.zip › jcla70339-sup-0007-Supinfo7@Supplementary Material2_rs2133314984.pdf]

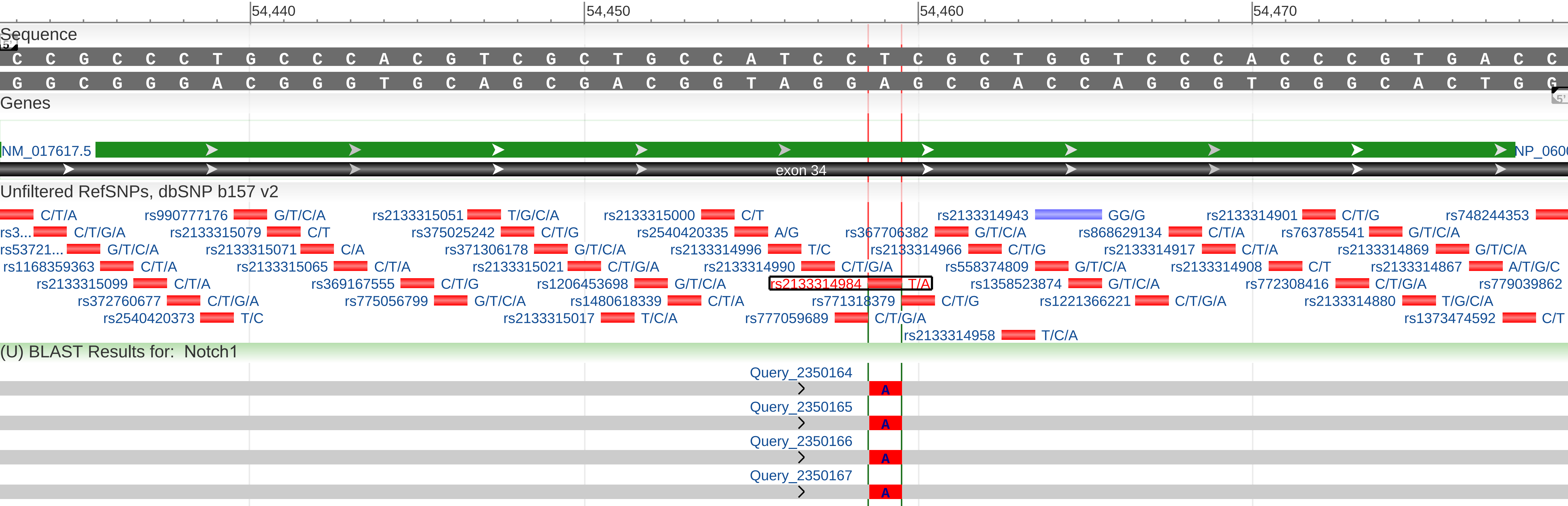
54,440
54,450
54,460
54,470
Sequence
C C G C C C T G C C C A C G T C G C T G C C A T C C T C G C T G G T C C C A C C C G T G A C C
G G C G G G A C G G G T G C A G C G A C G G T A G G A G C G A C C A G G G T G G G C A C T G G
Genes
NM_017617.5
NP_0600
exon 34
Unfiltered RefSNPs, dbSNP b157 v2
C/T/A
rs990777176 G/T/C/A
rs2133315051 T/G/C/A
rs2133315000 C/T
rs2133314943 GG/G
rs2133314901 C/T/G
rs748244353
rs3... C/T/G/A
rs2133315079 C/T
rs375025242 C/T/G
rs2540420335 A/G
rs367706382 G/T/C/A
rs868629134 C/T/A
rs763785541 G/T/C/A
rs53721... G/T/C/A
rs2133315071 C/A
rs371306178 G/T/C/A
rs2133314996 T/C
rs2133314966 C/T/G
rs2133314917 C/T/A
rs2133314869 G/T/C/A
rs1168359363 C/T/A
rs2133315065 C/T/A
rs2133315021 C/T/G/A
rs2133314990 C/T/G/A
rs558374809 G/T/C/A
rs2133314908 C/T
rs2133314867 A/T/G/C
rs2133315099 C/T/A
rs369167555 C/T/G
rs1206453698 G/T/C/A
rs2133314984 T/A
rs1358523874 G/T/C/A
rs772308416 C/T/G/A
rs779039862
rs372760677 C/T/G/A
rs775056799 G/T/C/A
rs1480618339 C/T/A
rs771318379 C/T/G
rs1221366221 C/T/G/A
rs2133314880 T/G/C/A
rs2540420373 T/C
rs2133315017 T/C/A
rs777059689 C/T/G/A
rs1373474592 C/T
rs2133314958 T/C/A
(U) BLAST Results for: Notch1
Query_2350164
A
Query_2350165
A
Query_2350166
A
Query_2350167
A
