# Supplementary material for: Analysis of Notch1 and Notch3 Signaling Pathway Components in Benign Prostatic Hyperplasia and Prostate Cancer Patients
Source: J Clin Lab Anal. 2026 Aug 18;40(19):e70339. doi: 10.1002/jcla.70339 (PMC13483564; doi:10.1002/jcla.70339)
Supplement: Supplementary file 1 [file JCLA-40-e70339-s001.zip › jcla70339-sup-0008-Supinfo8@Supplementary Material3_rs577451694.pdf]

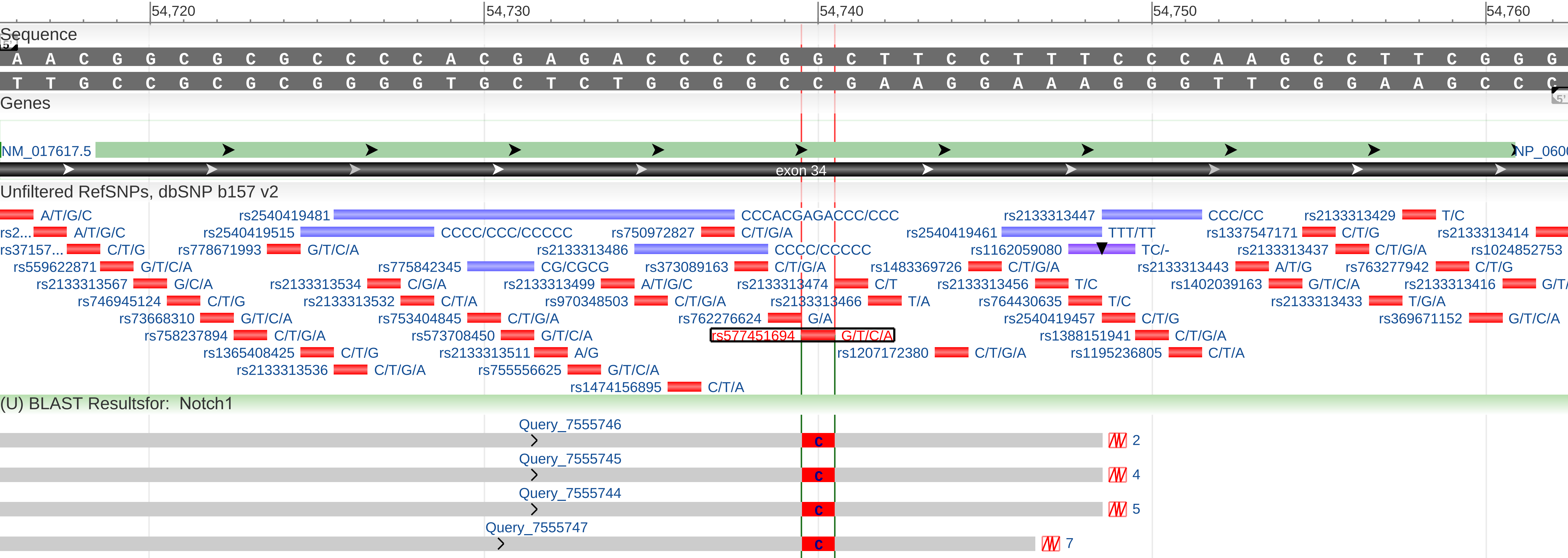

54,720
54,730
54,740
54,750
54,760
Sequence
A A C G G C G C G C C C C A C G A G A C C C C G G C T T C C T T T C C C A A G C C T T C G G G
T T G C C G C G C G G G G T G C T C T G G G G C C G A A G G A A A G G G T T C G G A A G C C C
Genes
NM_017617.5
NP_0600
exon 34
Unfiltered RefSNPs, dbSNP b157 v2
A/T/G/C
rs2540419481
CCCACGAGACCC/CCC
rs2133313447
CCC/CC
rs2133313429
T/C
rs2...
A/T/G/C
rs2540419515
CCCC/CCC/CCCCC
rs750972827
C/T/G/A
rs2540419461
TTT/TT
rs1337547171
C/T/G
rs2133313414
rs37157...
C/T/G
rs778671993
G/T/C/A
rs2133313486
CCCC/CCCCC
rs1162059080
TC/-
rs2133313437
C/T/G/A
rs1024852753
rs559622871
G/T/C/A
rs775842345
CG/CGCG
rs373089163
C/T/G/A
rs1483369726
C/T/G/A
rs2133313443
A/T/G
rs763277942
C/T/G
rs2133313567
G/C/A
rs2133313534
C/G/A
rs2133313499
A/T/G/C
rs2133313474
C/T
rs2133313456
T/C
rs1402039163
G/T/C/A
rs2133313416
rs746945124
C/T/G
rs2133313532
C/T/A
rs970348503
C/T/G/A
rs2133313466
T/A
rs764430635
T/C
rs2133313433
T/G/A
rs73668310
G/T/C/A
rs753404845
C/T/G/A
rs762276624
G/A
rs2540419457
C/T/G
rs369671152
G/T/C/A
rs758237894
C/T/G/A
rs573708450
G/T/C/A
rs577451694
G/T/C/A
rs1388151941
C/T/G/A
rs1365408425
C/T/G
rs2133313511
A/G
rs1207172380
C/T/G/A
rs1195236805
C/T/A
rs2133313536
C/T/G/A
rs755556625
G/T/C/A
rs1474156895
C/T/A
(U) BLAST Resultsfor: Notch1
Query_7555746
c
2
Query_7555745
c
4
Query_7555744
c
5
Query_7555747
c
7
