# Supplementary material for: Analysis of Notch1 and Notch3 Signaling Pathway Components in Benign Prostatic Hyperplasia and Prostate Cancer Patients
Source: J Clin Lab Anal. 2026 Aug 18;40(19):e70339. doi: 10.1002/jcla.70339 (PMC13483564; doi:10.1002/jcla.70339)
Supplement: Supplementary file 1 [file JCLA-40-e70339-s001.zip › jcla70339-sup-0009-Supinfo9@Supplementary Material4_rs746192089.pdf]

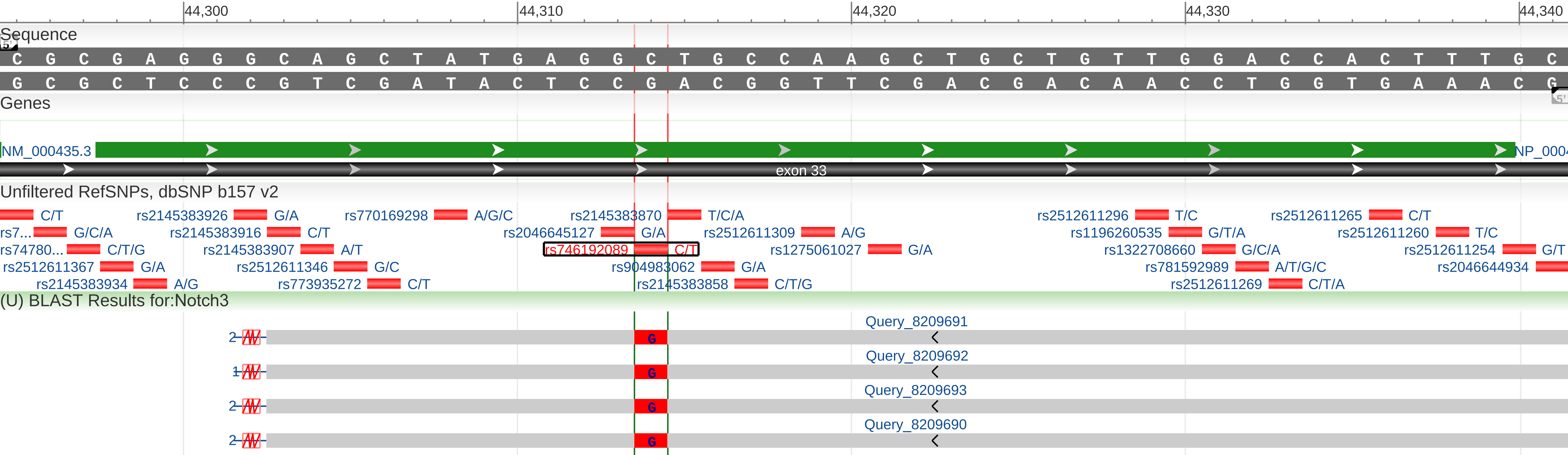

44,300
44,310
44,320
44,330
44,340
Sequence
C G C G A G G G C A G C T A T G A G G C T G C C A A G C T G C T G T T G G A C C A C T T T G C
G C G C T C C C G T C G A T A C T C C G A C G G T T C G A C G A C A A C C T G G T G A A A C G
Genes
NM_000435.3
NP_000
exon 33
Unfiltered RefSNPs, dbSNP b157 v2
C/T
rs2145383926 G/A
rs770169298 A/G/C
rs2145383870 T/C/A
rs2512611296 T/C
rs2512611265 C/T
rs7... G/C/A
rs2145383916 C/T
rs2046645127 G/A
rs2512611309 A/G
rs1196260535 G/T/A
rs2512611260 T/C
rs74780... C/T/G
rs2145383907 A/T
rs746192089 C/T
rs1275061027 G/A
rs1322708660 G/C/A
rs2512611254 G/T
rs2512611367 G/A
rs2512611346 G/C
rs904983062 G/A
rs781592989 A/T/G/C
rs2046644934
rs2145383934 A/G
rs773935272 C/T
rs2145383858 C/T/G
rs2512611269 C/T/A
(U) BLAST Results for:Notch3
Query_8209691
2
G
Query_8209692
1
G
Query_8209693
2
G
Query_8209690
2
G
