# Supplementary material for: Analysis of Notch1 and Notch3 Signaling Pathway Components in Benign Prostatic Hyperplasia and Prostate Cancer Patients
Source: J Clin Lab Anal. 2026 Aug 18;40(19):e70339. doi: 10.1002/jcla.70339 (PMC13483564; doi:10.1002/jcla.70339)
Supplement: Supplementary file 1 [file JCLA-40-e70339-s001.zip › jcla70339-sup-0010-Supinfo10@Supplementary Material5_rs1044009.pdf]

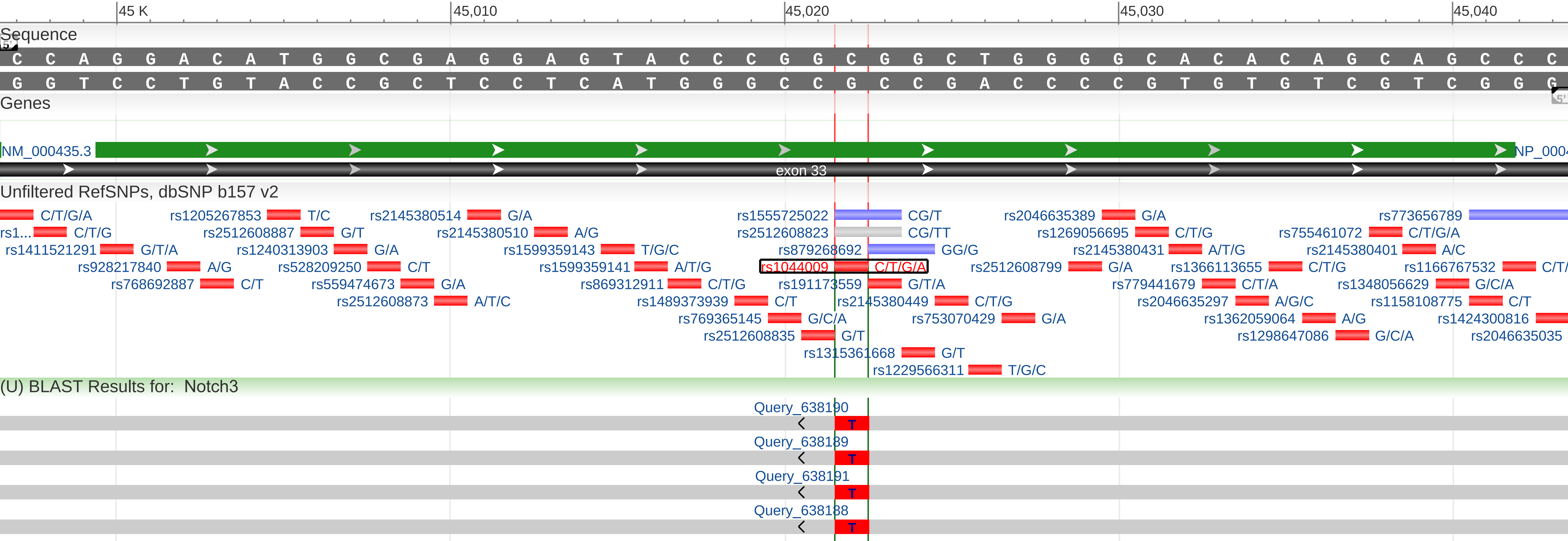

45 K
45,010
45,020
45,030
45,040
Sequence
C C A G G A C A T G G C G A G G A G T A C C C G G C G G C T G G G G C A C A C A G C A G C C C
G G T C C T G T A C C G C T C C T C A T G G G C C G C C G A C C C C G T G T G T C G T C G G G
Genes
NM_000435.3
NP_0004
exon 33
Unfiltered RefSNPs, dbSNP b157 v2
C/T/G/A
rs1205267853 T/C
rs2145380514 G/A
rs1555725022 CG/T
rs2046635389 G/A
rs773656789
rs1... C/T/G
rs2512608887 G/T
rs2145380510 A/G
rs2512608823 CG/TT
rs1269056695 C/T/G
rs755461072 C/T/G/A
rs1411521291 G/T/A
rs1240313903 G/A
rs1599359143 T/G/C
rs879268692 GG/G
rs2145380431 A/T/G
rs2145380401 A/C
rs928217840 A/G
rs528209250 C/T
rs1599359141 A/T/G
rs1044009 C/T/G/A
rs2512608799 G/A
rs1366113655 C/T/G
rs1166767532 C/T/
rs768692887 C/T
rs559474673 G/A
rs869312911 C/T/G
rs191173559 G/T/A
rs779441679 C/T/A
rs1348056629 G/C/A
rs2512608873 A/T/C
rs1489373939 C/T
rs2145380449 C/T/G
rs2046635297 A/G/C
rs1158108775 C/T
rs769365145 G/C/A
rs753070429 G/A
rs1362059064 A/G
rs1424300816
rs2512608835 G/T
rs1298647086 G/C/A
rs2046635035
rs1315361668 G/T
rs1229566311 T/G/C
(U) BLAST Results for: Notch3
Query_638190
T
Query_638189
T
Query_638191
T
Query_638188
T
